# Special Issue “Recent Advances in Wound Healing: 2nd Edition”

**DOI:** 10.3390/ijms27188417

**Published:** 2026-09-21

**Authors:** Carmen Curutiu, Alina Maria Holban

**Affiliations:** 1 Department of Microbiology, Faculty of Biology, University of Bucharest, Splaiul Independentei 91–95, 050095 Bucharest, Romania; alina.m.holban@bio.unibuc.ro; 2 Research Institute of the University of Bucharest-ICUB, 050663 Bucharest, Romania

Wound healing represents one of the most complex processes in the body and subjects the organism to the following sequential stages: hemostasias, inflammation, proliferation, and remodeling [1]. Many cells in the body (platelets, macrophages, neutrophils, fibroblasts, and epidermal cells) are involved in this well-coordinated process. Any change in external or internal factors can disrupt the process, leading to the appearance of chronic wounds, which results in incomplete healing, the recurrence of the infection process, and a continuous need for treatment [2,3]. This places an overwhelming burden on both the patient and the medical system [4,5]. Due to the complexity of the process, a multidisciplinary approach to diagnosis and treatment is often required. Since many aspects of chronic wound healing are still a long way from being completely understand, the aim of this collection of papers is to highlight the progress that currently being made by researchers in this field, as well as data from clinicians regarding the pathophysiology of wounds (acute or chronic) and the healing modalities currently available for these patients [6]. This Special Issue presents a valuable collection of papers. It includes two up-to-date reviews and seven original research articles, addressing a wide range of subjects, from analysis of bacterial species from wounds to recent developments of treatment solutions as new compounds for the fight against multidrug-resistant bacteria from chronic wound biofilms; the investigation of growth factors; and other new biologic wound products, skin substitutes, or interactive dressings.

Hidradenitis suppurativa (HS) is a severe chronic inflammatory disease characterized by recurrent nodules, abscesses, and fistulas [7,8]. Although numerous studies have shown that HS lesions are colonized by diverse bacteria and that bacterial biofilms contribute to the persistence of the disease, there was little information about the relationship between the bacterial species, their virulence factors, antibiotic resistance, and clinical severity of the disease [9]. The study by Cucu et al. [Contribution 1] on patients with chronic hidradenitis suppurativa, most of whom had moderate or severe forms of the disease, showed the diverse etiology of the bacterial species involved. Several bacterial strains belonging to 20 different species were isolated from the lesions, the most common being *Staphylococcus epidermidis* (28%), *Staphylococcus aureus* (15%) and *Enterococcus faecalis* (11%). The analyzed strains presented a diverse arsenal of virulence factors (hemolysins, lecithinases and lipases), enzymes involved in tissue destruction, invasiveness and bacterial persistence. The study also showed that all isolated bacterial strains were able to form biofilms, an important aspect because biofilms protect bacteria from antibiotics and the immune system response, favoring the chronicity of infections and the maintenance of inflammation [10]. Genetic analysis of *S. aureus* strains revealed a high frequency of genes involved in bacterial adhesion and host colonization (clfA, fnbA, and fib), as well as genes encoding toxins and hemolysins. Also, approximately half of the analyzed strains presented the mecA gene, which is characteristic of methicillin-resistant staphylococci (MRSA), and some of them contained macrolide resistance genes (ermC), indicating significant potential for resistance to treatment. Thus, the authors demonstrated that the microbiota associated with hidradenitis suppurativa lesions is not represented only by passive colonizing bacteria but includes microorganisms capable of forming complex biofilms, expressing multiple virulence factors and presenting antibiotic resistance mechanisms. These characteristics can significantly contribute to the persistence of inflammation, recurrence of lesions and difficulty of treatment. The study shows the need for development of new therapies, oriented not only towards eliminating bacteria but also towards destroying biofilms and inhibiting virulence factors involved in the pathogenesis of hidradenitis suppurativa.

The important role of bacterial biofilms in the development of wound complication is also addressed by Schoberleitner et al. (2024) [Contribution 2] in a review in which the mechanisms by which silicone breast implants cause fibrosis are analyzed. The authors describe, in detail, the biological processes that occur after implant insertion. The implant is recognized as a foreign body, which triggers an inflammatory response characterized by the recruitment of macrophages, lymphocytes and fibroblasts [11,12]. Over time, fibroblasts differentiate into myofibroblasts and produce increased amounts of collagen and other components of the extracellular matrix, favoring the formation of the fibrous capsule. In parallel, bacteria from the skin microbiota, especially *Staphylococcus epidermidis*, can adhere to the implant surface and form biofilms [13]. These structures protect microorganisms from the immune response and antibiotics, maintaining a chronic inflammation that amplifies the fibrotic process and favors capsular contracture. In the review, numerous preventive and therapeutic strategies are presented and evaluated. These include surgical measures to reduce bacterial contamination, the use of antiseptic and antibiotic solutions, and the development of new implant materials and surfaces with antimicrobial properties. Special polyurethane-based coatings; anti-adherent polymers; silver, zinc or titanium nanoparticles; biomimetic materials and other technologies capable of preventing bacterial adhesion and biofilm formation [14] are discussed. Antifibrotic and anti-inflammatory drugs, such as pirfenidone, halofuginone and dexamethasone, which have shown the ability to reduce fibrosis in experimental models, are also analyzed [15,16,17]. In conclusion, the authors emphasize that capsular fibrosis is the result of a complex interaction between the immune response to the foreign body and the persistence of microbial biofilms. Reducing bacterial adherence, preventing biofilm formation, and controlling the inflammatory and fibrotic response are essential directions for improving clinical outcomes [18].

A potential strategy for the treatment of bacterial infections associated with chronic wounds was proposed by Bahamondez-Cañas et al. (2026) [Contribution 3]. Building on the traditional use of medicinal plant *Buddleja globosa* (BG), which is recognized for its antimicrobial, anti-inflammatory, and regenerative properties [19], the authors developed four polymeric scaffold formulations with increasing concentrations of BG extract (BG1-BG4). These scaffolds were produced by lyophilization and formulated using gelatin, chitosan, and hyaluronic acid. The materials were designed for the treatment of infected wounds, particularly those colonized by mixed biofilms of *Pseudomonas aeruginosa* and *Staphylococcus aureus*, two bacterial species frequently found in chronic wounds and well known for their ability to form biofilms resistant to conventional therapies [20]. The results demonstrated that all BG-containing scaffolds reduced bacterial viability, with antimicrobial activity increasing proportionally to the extract concentration. However, BG2 exhibited the best overall performance, accelerating wound closure and promoting complete re-epithelialization. The authors concluded that the therapeutic efficacy of *Buddleja globosa*-loaded scaffolds depends not only on antimicrobial activity but also on the balance between extract concentration and the structural properties of the biomaterial. These findings support the potential use of *Buddleja globosa* extract-loaded scaffolds as an innovative strategy for the management of infected chronic wounds.

As a mechanism contributing to delayed wound healing, Wang et al. (2024) [Contribution 4] hypothesized that endothelial dysfunction—namely, impairment of the cells lining blood vessels—represents a key factor underlying delayed tissue repair. This hypothesis was based on previous observations indicating that animals subjected simultaneously to radiation exposure and cutaneous trauma develop a substantially more severe clinical course than those exposed to either injury alone [21]. To investigate this mechanism, the researchers employed a murine model in which mice received total body irradiation followed by induction of a skin wound. Survival, weight loss, and wound closure rates were evaluated, together with multiple biomarkers of inflammation and endothelial dysfunction, in both blood and injured tissues 3, 7, and 14 days after irradiation. Histological analyses were also conducted to assess the formation of granulation tissue, myofibroblast abundance, and collagen deposition, all of which are essential components of normal wound repair. The findings showed that irradiated and wounded mice exhibited higher mortality, greater weight loss, and significantly delayed wound healing compared with animals subjected only to wounding or irradiation. At the molecular level, more intense and persistent local and systemic inflammation was observed, accompanied by substantial alterations in endothelial injury markers. Furthermore, granulation tissue formation was delayed, myofibroblast numbers were reduced, and collagen deposition occurred more slowly and in a disorganized manner. The authors also identified dysregulation of the TGF-β signaling pathway, a major regulator of tissue regeneration and extracellular matrix formation. In conclusion, the study demonstrated that endothelial dysfunction and impaired TGF-β signaling are major mechanisms through which combined radiation and skin injuries delay healing and worsen clinical outcomes. The authors suggested that therapies aimed at protecting vascular endothelial cells or restoring TGF-β signaling could represent promising approaches for the treatment of patients affected by such complex injuries.

Elevated oxidative stress is another hallmark of chronic wounds and occurs when the production of reactive oxygen species exceeds the antioxidant capacity of the organism, as commonly observed in diabetic wounds and vascular ulcers [22]. This imbalance impairs healing through several mechanisms, including persistence of the inflammatory phase, local hypoxia, activation of matrix metalloproteinases that degrade extracellular matrix components and newly formed collagen, and induction of cellular senescence/apoptosis [23]. In this context, compounds with strong antioxidant properties may provide valuable therapeutic benefits. Jeskowiak-Kossakowska et al. (2025) [Contribution 5] investigated the therapeutic potential of three oil emulsions derived from transgenic flax varieties (M, B, and MB) and a linseed emulsion prepared from conventional NIKE linseed oil. These oils are rich in unsaturated fatty acids, phytosterols, and phenolic compounds with established antioxidant and anti-inflammatory activities. To evaluate their biological effects, the authors conducted a series of in vitro experiments using human dermal fibroblasts, keratinocytes, endothelial cells, and cutaneous tumor cell lines. Cellular viability, proliferation, migration (using a wound-healing model), cell-cycle distribution, apoptosis, necrosis, reactive oxygen species (ROS) production, and potential genotoxic effects on DNA were assessed. Hydrogen peroxide-induced oxidative stress models were additionally employed to evaluate the protective capacity of the emulsions against oxidative damage. The results demonstrated that all flax oil emulsions were safe for normal skin cells and did not exhibit significant cytotoxicity. Importantly, they did not stimulate the proliferation of skin tumor cells. All formulations promoted the proliferation and migration of normal cells involved in tissue regeneration, indicating beneficial effects on wound healing. A particularly significant finding was their strong antioxidant activity, as all emulsions reduced ROS levels and protected cellular DNA against oxidative damage. The authors concluded that emulsions derived from transgenic flax oils possess important regenerative, antioxidant, and genome-protective properties. They suggested that these products may be useful both for accelerating wound repair and for the care of skin affected by chronic inflammatory conditions. The most effective formulation was characterized by a high stigmasterol content combined with adequate levels of polyphenolic compounds and unsaturated fatty acids, particularly oleic and linoleic acids, which appear to act synergistically to reduce oxidative stress and promote cellular regeneration [24].

Recognizing that normal wound healing is often impaired by persistent inflammation and fibroblast dysfunction [25], in this context, Ceriani et al. (2026) [Contribution 6] investigated the role of Pannexin-1 (Panx1) channels, which are involved in extracellular ATP release and regulation of inflammatory responses. Because probenecid (PBN), a drug used in the treatment of gout, inhibits these channels, the authors investigated whether Panx1 inhibition would affect the regenerative actions of βFGF, a growth factor known to stimulate wound healing [26]. Cultured human neonatal dermal fibroblasts were treated with βFGF in the presence or absence of probenecid, and key regenerative processes, including fibroblast migration, cellular proliferation, expression of extracellular matrix remodeling genes (COL1A1, COL3A1, FN1, and VEGFA), extracellular ATP release, intracellular calcium-dependent signaling, and Panx1 protein expression and localization, were evaluated. The results demonstrated that Panx1 inhibition by probenecid did not significantly interfere with the beneficial effects of βFGF on fibroblast migration and proliferation. Likewise, most βFGF-induced changes in gene expression were maintained in the presence of probenecid. βFGF also reduced extracellular ATP release and transiently altered calcium signaling without affecting Panx1 expression levels or membrane localization. Pharmacological inhibition of Panx1 channels with probenecid is compatible with βFGF-induced regenerative responses in human dermal fibroblasts, so probenecid could serve as a useful therapeutic agent for limiting inflammation associated with chronic wounds while preserving the essential regenerative functions of fibroblasts.

Lu et al. (2024) [Contribution 7] addressed a critical challenge in regenerative medicine: the treatment of deep third-degree burns, which injure the skin and vasculature and typically require skin grafting. For treatment, a multidisciplinary approach is required, and skin grafting plays an essential role in restoring skin integrity and promoting healing [27,28]. The authors hypothesized that healing could be enhanced through the combination of three regenerative components: a pro-regenerative lipid mediator (PreM1) that promotes wound healing and present regenerative functions, an adipose tissue-derived microvascular fragment (MVF) rich in mesenchymal stem cells and endothelial cells, and a biomimetic amino acid-chitosan hydrogel (ACgel1) capable of serving as both a structural scaffold and a controlled-release system. To test this concept, the researchers developed a novel biomaterial, PreM1-MVFs-ACgel1, incorporating both PreM1 and MVFs into the hydrogel matrix. The formulation was applied to experimentally induced third-degree burns in mice. Outcomes included local PreM1 release, changes in the wound closure rate, re-epithelialization, neovascularization, and collagen deposition. Compared with individual components or partial combinations, the complete PreM1-MVFs-ACgel1 formulation demonstrated superior therapeutic efficacy. Thirteen days after injury, wounds treated with this composite system achieved approximately 97% closure and exhibited the smallest distance between newly formed epithelial edges. Furthermore, the formulation significantly enhanced angiogenesis and promoted collagen deposition and organization, both of which are essential for restoring skin structure and function. No adverse effects or behavioral changes were observed. The authors concluded that the combination of PreM1, MVFs, and ACgel1 exerts synergistic regenerative effects and markedly accelerates healing of severe burns. This platform was proposed as a potential alternative or therapeutic adjunct to conventional skin grafting, with the added advantage of reducing the need for healthy donor tissue.

Two studies examined the potential of the human amniotic membrane (hAM) as a regenerative biomaterial [29]. This membrane is the innermost layer of the placenta that presents distinctive structure and biological and physical characteristics that make it a highly biocompatible material for a variety of regenerative medicine applications [30,31]. In a comprehensive review, Sabbatini et al. (2025) [Contribution 8] synthesized current evidence regarding the structure, biological properties, and clinical applications of hAM. The authors discussed its composition and processing and preservation methods, including cryopreservation, lyophilization, and dehydration, as well as clinical outcomes reported in oral and periodontal surgery. Their analysis revealed that the amniotic membrane is rich in collagen, fibronectin, growth factors, cytokines, and stem cells, with significant regenerative potential. Owing to this composition, hAM promotes epithelial cell migration and differentiation [32], stimulates angiogenesis, reduces inflammation and fibrosis [33,34], limits scar formation, and exhibits antimicrobial and immunomodulatory properties [35,36,37]. An additional advantage is its exceptionally low immunogenicity, allowing its use with minimal risk of rejection. The review reported favorable outcomes in the treatment of gingival recessions, periodontal defects, bone defects following tumor excision, alveolar ridge preservation for dental implant placement, oral mucosal reconstruction, and medication-related osteonecrosis of the jaw (MRONJ). In most cases, hAM was associated with faster healing, improved re-epithelialization, reduced postoperative pain, enhanced aesthetic outcomes, and lower complication rates. The authors concluded that the human amniotic membrane represents a highly valuable biomaterial for regenerative oral surgery because it combines favorable mechanical characteristics with potent biological activity. It can serve simultaneously as a structural scaffold and as a source of bioactive molecules that promote tissue repair.

Considering that conventional preservation techniques, particularly dehydration, may alter the tissue architecture and reduce biologically active components, Harmon et al. (2024) [Contribution 9] investigated the potential of a hypothermically stored amniotic membrane (HSAM) as useful regenerative material [38]. To evaluate this approach, HSAM was compared with both unprocessed amniotic membrane (uAM) and dehydrated amniotic membrane (dAM). Tissue structure and thickness were assessed by histology and electron microscopy, mechanical properties were evaluated through tensile testing, degradation was examined in an experimental model simulating the chronic wound environment, and extracellular matrix proteins and growth factors were analyzed. In addition, the ability of HSAM to support fibroblast attachment and proliferation was investigated. The results demonstrated that HSAM retained the architecture and thickness of native amniotic membrane more effectively than dehydrated tissue, which became compacted and lost characteristic structural features. HSAM also exhibited mechanical properties and degradation resistance comparable to those of native amniotic membrane for up to 17 days under conditions resembling wound exudate. Immunohistochemical analyses confirmed the preservation of important regenerative molecules, including type I and III collagen, hepatocyte growth factor (HGF), IGF-1, and TGF-β1. In cell culture experiments, HSAM supported attachment, proliferation, and invasion of both human and murine fibroblasts, demonstrating its functionality as an active biological scaffold for tissue regeneration. The authors concluded that hypothermic storage effectively preserves both the structural and biological properties of native amniotic membrane. HSAM acts as an efficient scaffold for cellular growth, resists degradation in wound-like environments, and retains critical factors involved in tissue repair. These findings support its use as an advanced biological material for the treatment and management of both chronic and acute wounds.

This Special Issue highlights the remarkable diversity of mechanisms involved in wound healing and underscores the complexity of therapeutic strategies and the multidisciplinary approaches that may be employed to achieve success in wound management.
